# Ocular biometric characteristics in young Saudi adults across varying degrees of myopia

**DOI:** 10.7717/peerj.21767

**Published:** 2026-10-01

**Authors:** Saif Hassan Alrasheed, Azzam Abdulaziz Alharbi, Naveen Kumar Challa, Saleh Alshammeri, Abdelaziz Mohamed Elmadina, Bandar M. Alenezi, Fahad Almoqbel, Majid A. Moafa

**Affiliations:** 1 Department of Optometry, College of Applied Medical Sciences, Qassim University, Buraydah, Qassim, Saudi Arabia; 2 Department of Optometry, College of Applied Medical Sciences, King Saud University, Riyadh, Saudi Arabia

**Keywords:** Anterior chamber angle, Refractive error, Young adult, Myopia, Clinical feature

## Abstract

**Background:**

High myopia significantly increases the risk of serious ocular complications. The purpose of this study was to characterize ocular biometric variations across different degrees of myopia in young Saudi adults and to identify biometric parameters most strongly influenced by refractive severity.

**Methods:**

In this cross-sectional study, 163 eyes from individuals aged 18–31 years with myopia ranging from −1.00 to −11.00 D were evaluated at the university medical clinics. Anterior segment parameters such as white-to-white (WTW) corneal diameter, anterior chamber depth (ACD), anterior chamber volume (ACV), anterior chamber angle (ACA), and central corneal thickness (CCT) were measured using the Pentacam, axial length (AXL) was assessed with the IOL Master. Intraocular pressure (IOP) was measured with a non-contact tonometer. Finally, AXL, anterior chamber parameters, WTW, CCT and IOP were compared among the low, moderate, and high myopic groups.

**Results:**

Myopia severity showed a statistically significant positive correlation with AXL, WTW corneal diameter, ACD, ACV, CCT, and IOP (*p* < 0.05). AXL demonstrated a strong, statistically significant positive correlation with spherical equivalent (r = 0.70, *p* < 0.01). In contrast, ACA remained relatively stable across myopia groups. Multivariate analysis of covariance revealed that myopia severity significantly influenced AXL (F(2,156) = 8.52, *p* < 0.01), WTW corneal diameter (F(2,156) = 5.22, *p* < 0.01), ACV (F(2,156) = 7.94, *p* < 0.01), CCT (F(2,156) = 15.84, *p* < 0.01), and IOP (F(2,156) = 12.83, *p* < 0.01) after adjusting for age and gender. ACD showed modest increases across myopia groups with a borderline non-significant association after adjustment (F(2,156) = 2.93, *p* = 0.06), whereas ACA remained relatively stable and was not significantly affected by myopia severity (F(2,156) = 0.62, *p* = 0.54).

**Conclusion:**

Myopia severity in young adults is associated with significant structural changes, primarily AXL elongation and only minimal enlargement of anterior segment dimensions particularly the ACD and ACA, suggesting that posterior expansion predominates over anterior. These biometric patterns have important implications for refractive surgery planning, glaucoma risk assessment, and monitoring of myopia progression.

## Introduction

Uncorrected refractive errors such as hyperopia, myopia and astigmatism are considered the leading causes of global visual impairment (Holden et al., 2016; Alrasheed & Alghamdi, 2024). Myopia affects about 2.6 billion persons worldwide, and recent estimates suggest that its global prevalence will increase to about 4.8 billion individuals by 2050 (Holden et al., 2016). Previous studies (Alrasheed et al., 2026; Eppenberger et al., 2024; Alrasheed, Naidoo & Clarke-Farr, 2016) have shown that high myopia significantly increases the risk of ocular complications such as glaucoma, cataracts, macular degeneration, and retinal detachment which may ultimately lead to severe visual impairment. Recent studies (Eppenberger et al., 2024; Alotaibi et al., 2025) reported that the risk of ocular complications rises with increasing uncorrected myopia, which is defined as more than −0.50 D, while high myopia (>−6.00 D) is commonly connected with pathological changes that can cause blindness. Measurement of ocular biometric characteristics particularly white-to-white (WTW) corneal diameter, anterior chamber parameters (anterior chamber angle (ACA), anterior chamber depth (ACD), and anterior chamber volume (ACV)), and axial length (AXL) consider very important for accurate diagnosis planning for ocular surgery (Alotaibi et al., 2025; Sedaghat et al., 2017; Wei et al., 2021).

ACD is measured from the posterior corneal surface to the anterior lens surface, and ACA, formed between the cornea and iris, are key parameters in evaluating glaucoma risk, assessing aqueous outflow, and planning intraocular lens and some cataract surgery procedures (Alotaibi et al., 2025; Ning et al., 2019). ACV is defined as the aqueous-filled space between the posterior corneal surface and the anterior iris and lens complex, varies with age, degree of myopia, and other ocular parameters (Baumeister & Kohnen, 2018), while intraocular pressure (IOP), closely related to anterior chamber structure, is essential factor in glaucoma risk assessment and may be affected by high refractive error and change in the corneal parameters (Jonas et al., 2017).

Earlier studies (Alotaibi et al., 2025; Xu et al., 2021) have demonstrated positive relationship between WTW corneal diameter and anterior chamber parameters, with larger corneal diameters generally associated with deeper ACD this may increase the risk of angle-closure glaucoma. In addition, IOP commonly effected by anterior chamber anatomy, remains essential factor in glaucoma evaluation and may be altered following refractive surgery or change in corneal thickness. Despite advances in anterior segment imaging, information on ocular biometric variations across different degrees of myopia remain limited, emphasizing the need to evaluate the impact of myopia severity on ocular biometry to better understand the ocular complications associated with high myopia. Based on previously reported biometric patterns in myopic eyes, we hypothesized that greater myopia is associated with increased AXL and changes in anterior chamber parameters. Accordingly, this study aimed to assess ocular biometric characteristics across different degrees of myopia in young Saudi adults.

## Materials and Methods

### Study design

The present study employed a prospective cross-sectional design and was conducted at the Optometry Clinic, Qassim University, Saudi Arabia. The study included young adults with varying degrees of myopia ranging from −1.00 to −11.00 diopter (D) who attended the eye clinic from February to December 2025.

### Sample size calculation

The sample size for the present study was calculated to detect the correlation between the ocular biometric parameters and the varying degree of myopia using the Fisher *z*-transformation method for correlation studies, as described by Hulley et al. (2013). Assuming a moderate expected correlation of r = 0.30, with a 95% confidence level (Z {α/2} = 1.96) and 80% power (Z {β} = 0.84), the minimum required sample size was approximately 148 participants. The sample size was adjusted to account for a predicted 10% non-participation rate, the final sample size was increased to 163 myopic subjects, recruited from the Optometry Clinics at the Medical City, Qassim University. For each participant, the more myopic eye was included in the final analysis (Armstrong, 2013).

### Inclusion and exclusion criteria

The inclusion criteria comprised adults with a confirmed clinical diagnosis of myopia who provided written informed consent to participate in the study. In the present study, myopia was defined as a spherical equivalent refractive error of ≥−1.00 D and was further classified as low (−1.00 to −3.00 D), moderate (−3.25 to −6.00 D), or high (>−6.25 D) myopia (Flitcroft et al., 2019). In participants with bilateral myopia, only one eye was included to avoid inter-eye correlation and violation of statistical independence. The more myopic eye was selected based on spherical equivalent refractive error because it represents the eye with greater axial elongation and more pronounced structural changes associated with myopia severity. Exclusion criteria consist of subjects with a history of ocular surgery or trauma, presence of ocular or systemic diseases affecting the anterior or posterior segment (such as glaucoma, cataract, corneal pathology, diabetes, or retinal disease). The study also excluded persons with present use of medications affecting IOP or accommodation, active ocular infection or inflammation, strabismus, a history of corneal trauma or refractive surgery, systemic diseases, or media opacity that could affect accurate biometric measurements.

### Ethical consideration

Ethical approval was obtained from the Qassim University Health Research Ethics Committee (Approval No. 21-10-03). The study followed the Declaration of Helsinki, and written informed consent was obtained from all participants included in the study. Participation was voluntary, and all information were collected confidentially and anonymized. Privacy was confirmed by transmission anonymized identification codes to all participants, restricting access to data to the research team only, and storing records securely without personal identifiers.

### Clinical examinations

The participants underwent ocular history assessment followed by a detailed eye investigation. Then the visual acuity of each participant was measured using a Snellen chart at distance. Refractive errors were assessed using a non-cycloplegic autorefractometer (NIDEK-1; NIDEK, Tokyo, Japan). The anterior eye segment was examined using slit-lamp biomicroscopy and the posterior segment was evaluated by direct ophthalmoscopy and 90-diopter fundus biomicroscopy. Following the comprehensive ocular investigations, eligible participants underwent detailed measurements using the OCULUS Pentacam HR (Oculus, Wetzlar, Germany). The assessed parameters included central corneal thickness (CCT), ACD, ACA, ACV, and WTW corneal diameter. The Pentacam HR is a widely used advanced imaging device that provides high-resolution anterior segment tomography including cornea parameters and accurate biometric measurements for comprehensive ocular assessment (Zhang et al., 2023). All Pentacam measurements were performed in a dimly lit room under standardized illumination conditions. Three consecutive scans were obtained for each eye by an experienced examiner. Scan quality was assessed using the Pentacam quality specification indicator, and only scans labeled “OK” were accepted for analysis. Measurements that did not meet the quality criteria were repeated until an acceptable scan was obtained. Among multiple acceptable scans, the image with the best centration and overall quality was selected for analysis.

AXL was measured using the IOLMaster 700 (Carl Zeiss, Jena, Germany), while IOP was assessed using a non-contact tonometer (Nidek Tonoref, Tokyo, Japan). All examinations were performed by a trained optometrist following standardized procedures to minimize measurement variability. Circadian rhythms are known to affect several eye parameters, including IOP and CCT; thus, all assessments were performed at constant time points (Barberán-Bernardos, Ariza-Gracia & Piñero, 2024). All measurements were conducted between 10:00 AM and 12:00 PM to reduce the influence of diurnal variation. This mid-morning window was selected to avoid early morning peaks in IOP, which typically occur upon awakening and gradually decline throughout the day. However, some degree of physiological variation may still occur within this 2-h interval (Syam, Mavrikakis & Liu, 2005).

### Statistical analysis

In the present study data were analyzed using IBM SPSS Statistics, version 25 (IBM Corp., Armonk, NY, USA). The Shapiro–Wilk test was used to check whether the data were normally distributed. Descriptive statistics were used to summarise ocular biometric measurements and expressed as mean and standard deviation. Relationship between degree of myopia (measured as spherical equivalent), AXL, ACV, ACD, ACA, and horizontal WTW corneal diameter, central CCT, and IOP were assessed using Pearson’s correlation coefficient. Correlations were classified as very weak (<0.1), weak (0.1–0.3), moderate (0.3–0.6), or strong (>0.6). Statistical significance was set at *p* < 0.05. Comparison of biometric parameters for three myopic grades were analysed using analysis of variance (ANOVA). Multivariate Analysis of Covariance (MANCOVA) was performed to see the effect of myopia on biometric parameters after controlling for confounders such age and gender.

A sensitivity analysis was conducted to evaluate the potential impact of myopia severity misclassification. Participants at the classification boundaries were reclassified using an alternative categorization approach, and all statistical analyses were repeated to assess the robustness of the primary findings.

## Results

The descriptive characteristics of the participants were summarized by myopia severity and gender (Table 1). Among the study cohort, 54 eyes were categorized as low myopic, 90 as moderate myopic, and 19 as high myopic. Each myopia group comprised both female and male participants. As expected, AXL and anterior segment dimensions tended to increase with increasing myopia severity. Other ocular parameters, including ACV, ACD, ACA, IOP, and CCT were also systematically evaluated across the groups.

**Table 1:** Descriptive statistics (mean ± SD) for ocular biometric parameters stratified by degree of myopia (low, moderate, and high) and gender.

Myopia	Gender	N	SE (Diopter)	AXL (mm)	WTW (mm)	ACV (mm^3^)	ACA (Degree)	ACD (mm)	IOP (mmHg)	CCT (µM)
Low	Female	32	−1.97 ± 0.76	24.16 ± 0.81	11.85 ± 0.34	184.78 ± 22.99	40.37 ± 3.54	3.08 ± 0.25	16.16 ± 2.70	551.78 ± 36.35
	Male	22	−2.05 ± 0.65	24.38 ± 0.83	12.05 ± 0.23	188.50 ± 16.69	39.47 ± 3.89	3.18 ± 0.24	18.05 ± 3.00	547.05 ± 37.47
	Total	**54**	**−2.00 ± 0.71**	**24.25 ± 0.82**	**11.93 ± 0.31**	**186.30 ± 20.57**	**40.00 ± 3.67**	**3.12 ± 0.25**	**16.93 ± 2.95**	**549.85 ± 36.53**
Moderate	Female	48	−4.43 ± 0.72	25.66 ± 1.00	12.06 ± 0.34	198.92 ± 14.00	39.88 ± 3.49	3.16 ± 0.18	18.98 ± 2.46	583.83 ± 30.18
	Male	42	−4.36 ± 0.75	25.63 ± 0.86	12.19 ± 0.28	197.00 ± 15.37	40.54 ± 3.52	3.23 ± 0.20	18.55 ± 2.92	576.29 ± 36.14
	Total	**90**	**−4.40 ± 0.73**	**25.65 ± 0.93**	**12.12 ± 0.32**	**198.02 ± 14.61**	**40.19 ± 3.50**	**3.19 ± 0.19**	**18.78 ± 2.68**	**580.31 ± 33.12**
High	Female	9	−7.42 ± 1.98	26.45 ± 1.88	11.65 ± 0.51	176.44 ± 31.45	39.47 ± 6.34	3.15 ± 0.31	19.00 ± 3.06	557.56 ± 43.13
	Male	10	−6.48 ± 0.48	27.29 ± 1.19	12.55 ± 0.49	207.00 ± 14.14	40.85 ± 7.18	3.43 ± 0.31	21.50 ± 1.78	604.80 ± 29.02
	Total	**19**	**−6.92 ± 1.45**	**26.89 ± 1.57**	**12.12 ± 0.67**	**192.53 ± 28.02**	**40.19 ± 6.64**	**3.30 ± 0.33**	**20.47 ± 2.63**	**582.42 ± 42.84**

**Note:**

SE, spherical equivalent; AXL, axial length; WTW, white to white; ACV, anterior chamber volume; ACA, anterior chamber angle; ACD, anterior chamber depth; IOP, intraocular pressure; CCT, central corneal thickness.

The Table 1 summarizes spherical equivalent (SE), AXL, horizontal WTW, ACV, ACA, ACD, IOP, and CCT. Values are reported separately for females, males, and the total sample within each myopia category to illustrate biometric differences across severity levels and between genders.

The biometric parameters, including AXL, WTW corneal diameter, ACV, ACA, ACD, and CCT, were compared across different grades of myopia (low, moderate, and high). Box plot analysis (Fig. 1) demonstrated a clear upward shift in AXL with increasing myopia severity, consistent with progressive ocular elongation. This visual trend was supported by one-way ANOVA (See Table 2), which revealed a highly significant difference in AXL among the three groups (*p* < 0.001). Median AXL increased from 24.25 mm in the low myopia group to 25.65 mm in the moderate group and reached 26.89 mm in the high myopia group. Furthermore, the interquartile range was notably wider in the high myopia group, indicating increased variability in axial elongation among individuals with more severe refractive error.

**Figure 1:**
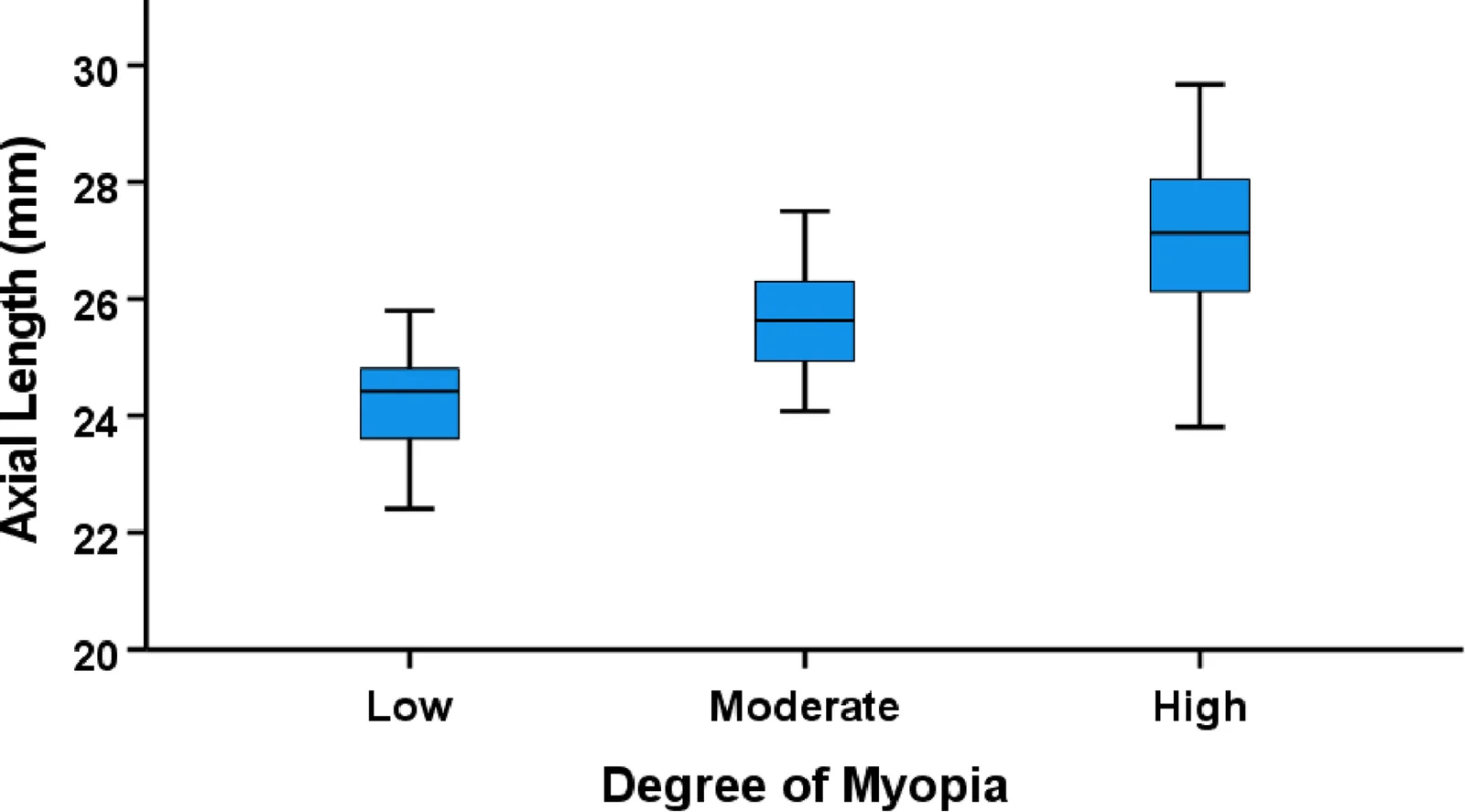
Boxplots showing axial length across low, moderate, and high degrees of myopia.

**Table 2:** Comparison of ocular biometric parameters across degrees of myopia using one-way ANOVA.

Parameter	Low myopia (mean ± SD)	Moderate myopia (mean ± SD)	High myopia (mean ± SD)	ANOVA *p*-value
AXL (mm)	24.25 ± 0.82	25.65 ± 0.93	26.89 ± 1.57	<0.01
WTW (mm)	11.93 ± 0.31	12.12 ± 0.32	12.12 ± 0.67	0.01
ACV (mm^3^)	186.30 ± 20.57	198.02 ± 14.61	192.53 ± 28.02	<0.01
ACA (°)	40.00 ± 3.67	40.19 ± 3.50	40.19 ± 6.64	0.961
ACD (mm)	3.12 ± 0.25	3.19 ± 0.19	3.30 ± 0.33	0.02
IOP (mm of Hg)	16.93 ± 2.95	18.78 ± 2.68	20.47 ± 2.63	<0.01
CCT (µM)	549.85 ± 36.53	580.31 ± 33.12	582.42 ± 42.84	<0.01

**Note:**

AXL, axial length; WTW, White-to-white; ACV, anterior chamber volume; ACA, anterior chamber angle; ACD, anterior chamber depth; IOP, intraocular pressure; CCT, central corneal thickness.

Visual inspection of the box plots (Fig. 2) suggested an upward shift in WTW corneal diameter (WTW; Fig. 2A) and central corneal thickness (CCT; Fig. 2B) with increasing myopia severity, with the clearest separation observed between the low and moderate myopia groups. These observations were supported by one-way ANOVA (Table 2), which demonstrated statistically significant differences among myopia groups for both WTW (*p* = 0.012) and CCT (*p* < 0.001). Pairwise comparisons indicated that mean WTW and CCT values were significantly higher in the moderate myopia group compared with the low myopia group (*p* 0.01). Whereas no statistically meaningful difference was detected between the moderate and high myopia groups. Notably, the high myopia group demonstrated a wider interquartile range for both parameters, indicating greater variability in WTW corneal diameter and CCT among individuals with severe myopia.

**Figure 2:**
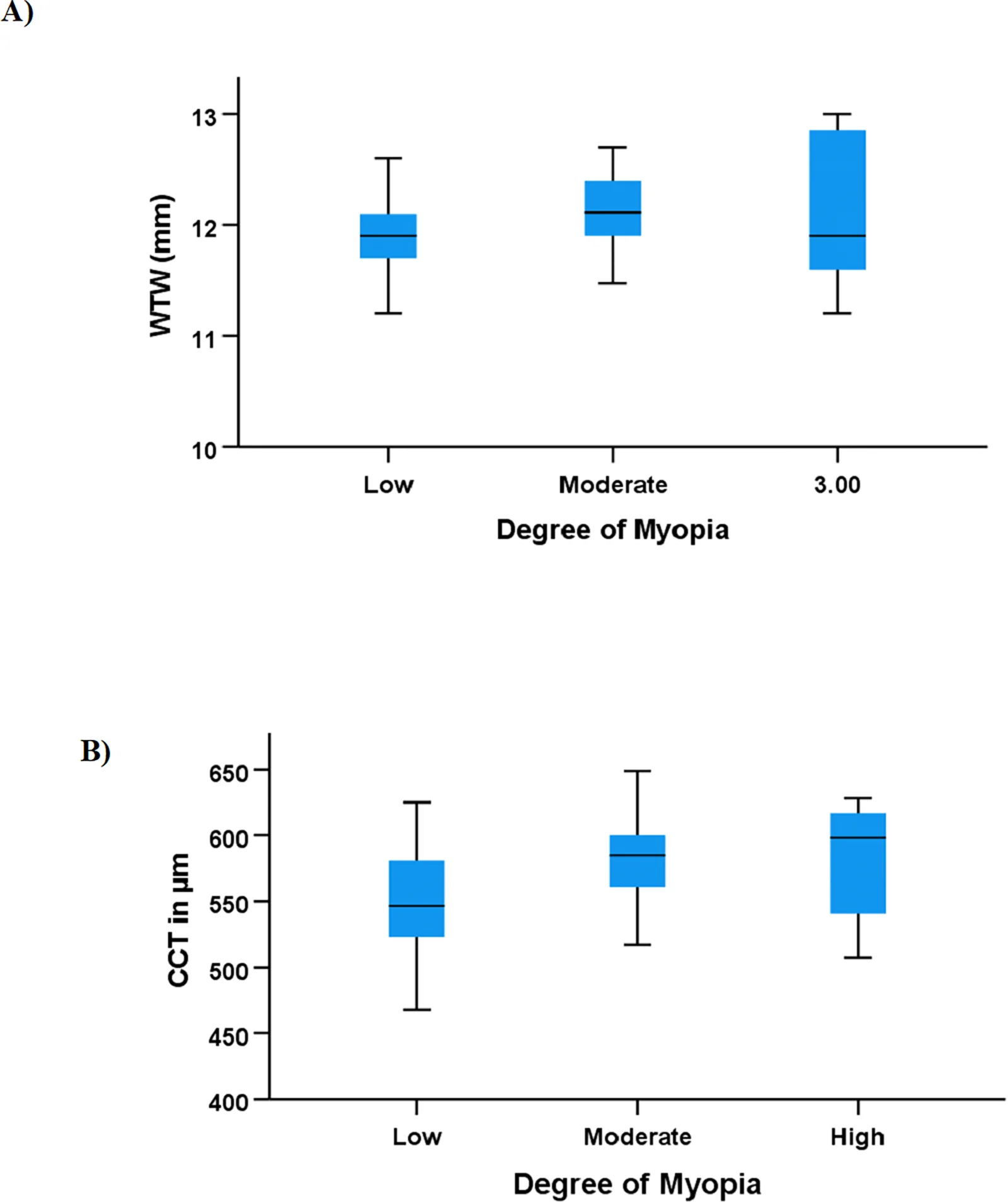
Boxplots showing (A) HWTW corneal diameter and (B) CCT across low, moderate, and high degrees of myopia.

Similarly, box plots illustrating anterior chamber parameters (Figs. 3A–3C) demonstrated an increasing trend in ACV and ACD with advancing myopia severity, with median values lowest in the low myopia group and highest in the high myopia group. One-way ANOVA (Table 2) confirmed significant group differences for ACV (*p* = 0.002) and ACD (*p* = 0.015). ACV and ACD values were significantly higher in the moderate myopia group compared with the low myopia group, whereas increases between the moderate and high myopia groups were less pronounced. In contrast, ACA exhibited comparable central tendencies across myopia groups, with no statistically significant difference observed (*p* = 0.961), although greater variability was evident among individuals with high myopia. Consistent with corneal findings, the high myopia group demonstrated increased dispersion in anterior chamber measurements, suggesting greater anatomical variability with increasing myopia severity.

**Figure 3:**
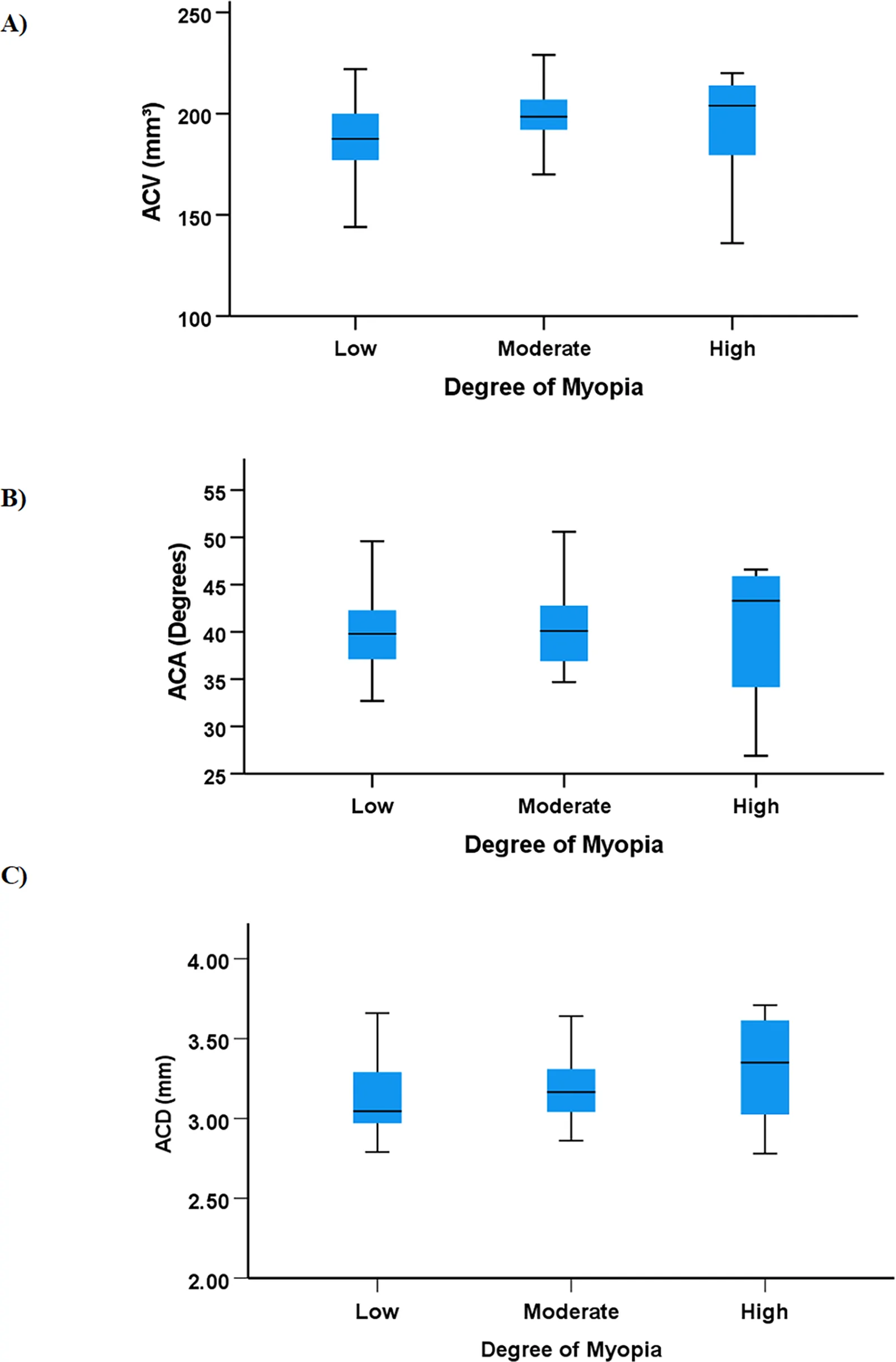
Boxplots showing (A) Anterior chamber volume (ACV), (B) anterior chamber angle (ACA), and (C) anterior chamber depth (ACD) across low, moderate, and high degrees of myopia.

The box plots for IOP (Fig. 4) demonstrated a clear upward shift in median and mean values with increasing myopia severity. This trend was supported by ANOVA (Table 2), which revealed a highly significant difference among the three myopia groups (*p* < 0.001). Mean IOP increased from 16.93 mmHg in the low myopia group to 18.78 mmHg in the moderate group and reached 20.47 mmHg in the high myopia group, indicating a progressive elevation of IOP with increasing axial elongation.

**Figure 4:**
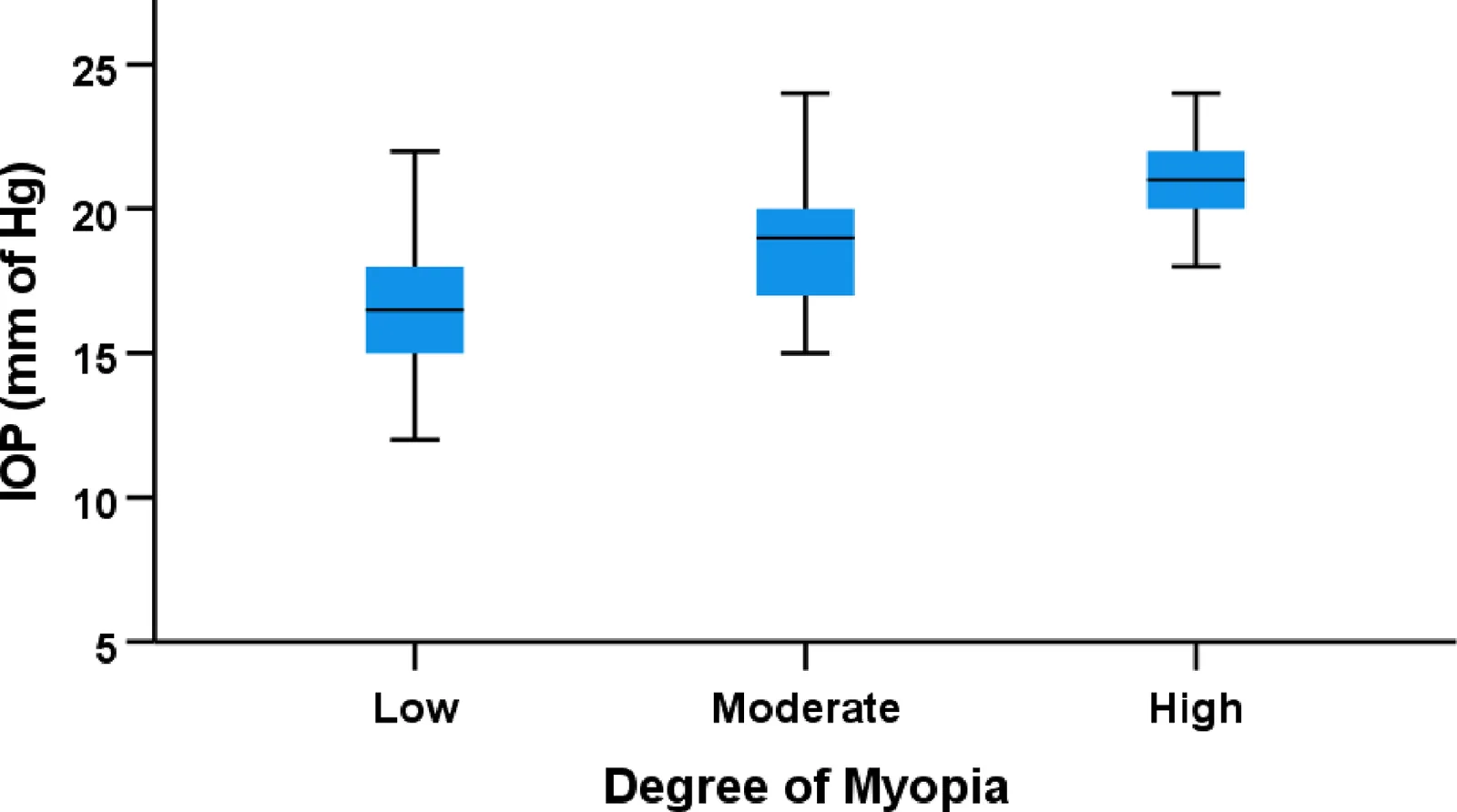
Boxplots showing intraocular pressure (IOP) across low, moderate, and high degrees of myopia.

Correlation analysis (Table 3) revealed that SE was strongly associated with AXL (r = 0.70, *p* < 0.01) and moderately correlated with IOP (r = 0.44, *p* < 0.01), CCT (r = 0.31, *p* < 0.01), ACD (ACD; r = 0.28, *p* < 0.01), ACV (r = 0.30, *p* < 0.01), and WTW (r = 0.20, *p* < 0.05). No significant correlation was observed between SE and ACA. Whereas, ACA was positively associated with ACV and ACD but showed a weak negative relationship with IOP. Moreover, WTW corneal diameter correlated positively with ACV, ACA, ACD, IOP, and CCT.

**Table 3:** Correlation coefficients between age and various ocular biometric variables.

Parameter	AGE	SE	AXL	WTW	ACV	ACA	ACD	IOP	CCT
AGE	1.00	0.12	0.21**	0.24**	0.23**	0.36**	0.18*	−0.13	−0.14
SE		1.00	0.70**	0.20*	0.30**	0.06	0.28**	0.44**	0.31**
AXL			1.00	0.45**	0.39**	0.27**	0.50**	0.42**	0.33**
WTW				1.00	0.59**	0.38**	0.54**	0.45**	0.48**
ACV					1.00	0.22**	0.47**	0.32**	0.30**
ACA						1.00	0.61**	−0.20	−0.06
ACD							1.00	0.28**	0.24**
IOP								1.00	0.65**
CCT									1.00

**Notes:**

* *p* < 0.05.

** *p* < 0.01.

AXL, axial length; WTW, white-to-white; ACV, anterior chamber volume; ACA, anterior chamber angle; ACD, anterior chamber depth; IOP, intraocular pressure; CCT, central corneal thickness.

Correlation coefficients between age and various ocular biometric variables, including SE, AXL, WTW corneal diameter, ACV, ACA, ACD, IOP, and CCT. Significant correlations are indicated as follows: *p* < 0.05 (*), *p* < 0.01 (**).

Analysis of covariance (ANCOVA) was performed to evaluate the effect of myopia severity on ocular biometric parameters while controlling age and gender. The overall corrected models were statistically significant for all evaluated variables, including AXL, WTW, ACV, ACA, ACD, IOP, and CCT (all *p* < 0.01), indicating that the combined influence of covariates and myopia grade explained a substantial proportion of variability in these parameters. A sensitivity analysis using an alternative classification of myopia severity yielded findings comparable to those of the primary analysis, with no meaningful changes in the direction or statistical significance of the observed associations, indicating that the results were robust to potential misclassification.

After adjustment, degree of myopia remained a highly significant independent predictor of AXL (F = 8.52, *p* < 0.001), confirming progressive axial elongation with increasing refractive severity. Significant effects of myopia grade were also observed for WTW (F = 5.22, *p* < 0.01), ACV (F = 7.94, *p* = 0.001), IOP (F = 12.83, *p* < 0.01), and CCT (F = 15.84, *p* < 0.01), demonstrating that differences in corneal dimensions, ACV, IOP, and CCT across myopia groups persisted even after controlling for demographic confounders. In contrast, no statistically significant association was found between degree of myopia and ACA (*p* = 0.54). While the association with ACD approached statistical significance after adjustment for age and gender (*p* = 0.06), the attenuation of the effect compared with the unadjusted analysis suggests potential confounding by these demographic factors (age and gender), as presented in Table 4. These findings suggest that while axial elongation and several anterior segment parameters vary significantly with myopia severity, ACA appears relatively stable across refractive groups when adjusted for age and gender.

**Table 4:** Summary of MANCOVA examining the effect of myopia severity on ocular biometric parameters adjusted for age and gender.

Variable	Myopia degree F(2,156)	*p*	ηp^2^	Age F(1,156)	*p*	Gender F(1,156)	*p*
AXL	8.52	<0.01	0.42	7.09	<0.01	0.5	0.48
WTW	5.22	<0.01	0.06	11.45	<0.01	14.23	<0.01
ACV	7.94	<0.01	0.09	9.62	<0.01	1.12	0.29
ACA	0.62	0.54	0.01	21.05	<0.01	0.0	0.97
ACD	2.93	0.06	0.04	3.06	0.08	6.8	0.01
IOP	12.83	<0.01	0.14	4.74	0.03	3.06	0.08
CCT	15.84	<0.01	0.17	2.14	0.146	0.04	0.83

**Note:**

AXL, axial length; WTW, white-to-white; ACV, anterior chamber volume; ACA, anterior chamber angle; ACD, anterior chamber depth; IOP, intraocular pressure; CCT, central corneal thickness.

With respect to covariates, age demonstrated significant effects on multiple parameters, including AXL (*p* = 0.01), WTW (*p* < 0.01), ACV (*p* = 0.002), ACA (*p* < 0.01), and IOP (*p* = 0.03), indicating age-related structural changes in ocular biometry. Gender showed significant associations with WTW (*p* < 0.01) and ACD (*p* = 0.01), suggesting sex-related anatomical differences, while its influence on other parameters was not statistically significant, as shown in Table 4.

The proportion of variance explained by the models was highest for AXL (R^2^ = 0.45), followed by CCT (R^2^ = 0.18) and IOP (R^2^ = 0.18), highlighting strong explanatory power for axial elongation in relation to myopia severity and demographic factors. Values are presented as F statistics with corresponding *p*-values. Partial eta squared (ηp^2^) represents the effect size for degree of myopia. Bold *p*-values indicate statistical significance (*p* < 0.05).

## Discussion

This study provides a comprehensive characterization of ocular biometric variation across different grades of myopia in young Saudi adults. AXL demonstrated a clear stepwise increase from low to high myopia, with values ranging from 24.25 ± 0.82 mm in low myopia to 26.89 ± 1.57 mm in high myopia. This pattern aligns with the well-established role of axial elongation as the principal structural correlate with myopia and is consistent with reports from East Asian, European, African, and North American cohorts (Hulley et al., 2013; Armstrong, 2013; Flitcroft et al., 2019; Zhang et al., 2023; Barberán-Bernardos, Ariza-Gracia & Piñero, 2024; Syam, Mavrikakis & Liu, 2005; Dong et al., 2025; Chen et al., 2024; Liu et al., 2025; Tideman et al., 2018; Nilagiri et al., 2023; Jiang et al., 2023). The observed greater AXL variability in high myopia likely reflects the heterogeneity of advanced cases, where scleral remodelling differs markedly among individuals. Eyes with AXL exceeding 26 mm carry increased risk of posterior segment complications, such as retinal detachment and posterior staphyloma, and pose challenges for accurate IOL power calculation. Early identification of rapid axial elongation, particularly in younger adults, is therefore essential for guiding myopia-control strategies and precise surgical planning (Obinwanne, Naidoo & Mashige, 2024; Midorikawa et al., 2024). The present findings suggest that myopia severity is mainly associated with AXL enlargement, with minimal changes in anterior segment parameters, highlighting the dominance of posterior structural alterations and underscoring the clinical importance of AXL assessment in myopia prevention, management and surgical planning.

The present study demonstrated that anterior segment characteristics also varied systematically with myopia severity. WTW corneal diameter remained relatively stable between moderate and high myopia groups and also there is sex-based differences, with males exhibiting larger corneal diameters than females. This trend mirrors recent Saudi normative data obtained using Pentacam AXL Wave (Alotaibi et al., 2025) and aligns with findings from Chinese, Japanese, Indian, European, and African populations demonstrating larger corneal dimensions in males (Li, Wang & Liao, 2024; Zhang et al., 2022; Singh et al., 2019; Rüfer, Schröder & Erb, 2005; Alrasheed & Aldakhil, 2022; Iyamu & Osuobeni, 2012). Although exceptions have been reported (Iyamu & Osuobeni, 2012), the predominance of evidence supports meaningful sex-related ocular size differences that warrant consideration during refractive surgery planning and IOL calculation. The present study showed a modest increase in CCT with greater myopia severity, with the thickest corneas observed among high-myopic males. This contrasts with findings from Chinese and South Asian populations, where CCT commonly shows minimal change or decreases with increasing myopia severity (Zhang et al., 2026; Jiang et al., 2024; Panda, Mohapatra & Khuntia, 2025). The population-specific differences observed across above-mentioned studies may be attributed to variations in genetic background, environmental exposures, and ocular growth patterns among different ethnic groups. Additionally, differences in study populations and measurement techniques may contribute to the inconsistent associations reported between CCT and myopia severity. Numerous mechanisms may explain the findings of the present study. The association between CCT and AXL appears to vary across populations, and ocular elongation may be accompanied by compensatory changes in anterior segment structures. Increased stromal hydration, may altered corneal biomechanical properties, or differences in scleral stiffness in highly myopic eyes may reorganize biomechanical forces toward the cornea, resulting in increased thickness. Sex-related factors, such as variations in collagen composition and hormonal influences, may further contribute to the thicker CCT observed in males. Supporting this, study in Chinese have shown that eyes with AXL ≥26 mm exhibit significantly thicker CCT compared with shorter eyes, suggesting that extreme axial elongation may be associated with structural corneal adaptations (Moafa et al., 2025).

In the present study, anterior chamber depth and anterior chamber volume positively correlated with greater myopia severity, while the anterior chamber angle remained relatively unchanged. These findings align with previously published studies and are consistent with biometric trends reported in different ethnicities, where longer eyes typically exhibit larger and deeper anterior chambers (Zhang et al., 2026; Niknahad et al., 2025; Mashige & Oduntan, 2017). Within our cohort, high-myopic males exhibited the largest ACV and deepest ACD, a finding that parallels with established sex-related differences in ocular dimensions. Additionally, the present study showed that high IOP associated with myopia severity, reaching its highest levels among males with high myopia. This pattern is consistent with previous reports from Asian and Middle Eastern populations that associate moderate to high myopia with elevated IOP (Zhou et al., 2022; Hashemi et al., 2024). Although the causal relationship between IOP and myopia progression remains complex and multifactorial, the observed association warrants further investigation (Deng et al., 2024). Elevated IOP in myopic eyes may reflect biomechanical changes in scleral and lamina cribrosa integrity, potentially increasing susceptibility to glaucomatous damage. Longitudinal studies are needed to clarify whether elevated IOP contributes to axial elongation or merely correlates with it.

The correlation matrix revealed coherent ocular scaling patterns. Myopic spherical equivalent showed strong association with AXL and moderate associations with ACD, ACV, IOP, WTW, and CCT, indicating that more myopic eyes are not only longer but also possess proportionally larger anterior segments. AXL was significantly correlated with WTW, ACV, and ACD, consistent with structural coupling reported in diverse populations (Niknahad et al., 2025; Mashige & Oduntan, 2017; Marinescu et al., 2025; Fan, Huang & Huang, 2024; Dabasia et al., 2015). Notably, the positive correlation between WTW corneal diameter and CCT in this cohort contrasts with East Asian studies where larger corneal diameters often corresponds to thinner corneas (Gharaee et al., 2014). The relatively strong CCT–IOP correlation (r = 0.65) observed in this study exceeds values commonly reported and carries important clinical implications. Thicker corneas may cause overestimation of IOP, potentially obscuring true pressure levels and affecting glaucoma risk assessment. The use of non-contact tonometry (NCT) represents a limitation, particularly in myopic eyes where corneal properties may introduce measurement bias. High myopia is often associated with thinner corneas and altered biomechanics, which can affect NCT accuracy. However, the strong correlation between CCT and IOP in this study (r = 0.65) suggests that IOP measurements may be influenced by corneal thickness rather than reflecting true variations in IOP. Although our findings indicate that CCT influences IOP measurements in high myopic eyes, this study did not validate a specific pachymetry-based correction formula. Therefore, no particular correction approach can be recommended. Given that CCT–IOP relationships vary across populations, generalized correction formulae should be applied with caution, and further studies are needed to determine their clinical utility.

Multivariate analysis confirmed that AXL was the most strongly influenced structural parameter associated with myopia severity (F(2,156) = 8.52, *p* < 0.01), supporting previous literature identifying axial elongation as the primary structural correlate of refractive error. Myopia severity also significantly influenced WTW, ACV, CCT, and IOP after adjusting for age and gender. In contrast, ACD showed a borderline non-significant association *p* = (0.06), while ACA did not demonstrate significant differences across myopia groups. The borderline significance of ACD after adjustment for age and gender (*p* = 0.06) suggests that demographic factors may partially account for the observed association, underscoring the importance of considering potential confounding effects when interpreting this finding. Collectively, these findings support the concept of coordinated ocular structural changes in myopic eyes, with axial elongation representing the most prominent structural alteration associated with increasing myopia severity (Syam, Mavrikakis & Liu, 2005). A sensitivity analysis using an alternative classification of myopia severity produced results that were consistent with the primary analysis. The overall pattern of associations remained unchanged, indicating that the study findings were robust to potential misclassification of myopia severity.

The biometric profile of higher myopia characterized by greater AXL, deeper anterior chambers, increased ACV, elevated IOP, large and thicker corneas, reflects the complex interaction between posterior and anterior segment changes in high myopic eyes. Although ACA did not show significant variation between groups, this finding should be interpreted cautiously. The Pentacam relies on Scheimpflug imaging, which cannot directly visualize the scleral spur or angle recess, making it prone to measurement variation that can obscure subtle anatomical changes. Therefore, a stable ACA reading does not definitively rule out minor structural alterations. This limitation aligns with previous literature (Doyle et al., 2026) showing that Pentacam has lower sensitivity for subtle angle tracking compared to gonioscopy or anterior segment optical coherence tomography. These structural characteristics have important clinical implications. For refractive surgery, increased ACD and ACV enhance suitability for phakic intraocular lenses, while CCT variability requires individualized corneal safety thresholds. Importantly, AXL remains the most reliable parameter for monitoring myopia progression and minimizing postoperative refractive error in highly elongated eyes.

Although this study provides important insights, it has some limitations. First, refractive error was measured using an autorefractometer and IOP using non-contact tonometry rather than subjective refraction and Goldmann applanation tonometry, the gold standards. Although these methods improved efficiency and reduced examiner-dependent variability, autorefractometer may overestimate myopia in young adults, and non-contact tonometry is less accurate at higher IOP levels. In addition, the use of non-cycloplegic refraction may introduce bias due to unrelaxed accommodation, commonly causing a myopic shift in the measured refractive error. As a result, some individuals categorized as low myopes may be emmetropic. This would tend to overestimate the myopia and exaggerate its magnitude, with a greater effect expected in younger participants or those with active accommodation (Dabasia et al., 2015). However, in adult populations, accommodative effects are generally lower than in children, and previous study (Doyle et al., 2026; Khan et al., 2024) has reported relatively small mean differences between cycloplegic and non-cycloplegic refraction in young adults approximately −0.20 D. Cycloplegic refraction is time-consuming, may cause patient discomfort, and is less practical in large-scale or field studies; therefore, non-cycloplegic autorefraction is commonly used as a feasible alternative in epidemiological research (Kaur & Gurnani, 2026). Second, the study sample was derived from a single center in Saudi Arabia, which may limit external validity and generalizability to other ethnicities and geographic regions. Future studies involving more diverse populations and broader inclusion criteria are recommended to validate these findings. Third, the small sample size of the high myopia group (*n* = 19) compared to the moderate and low groups may limit statistical power and increase variability, as reflected by larger standard deviations. This may affect the stability and reliability of estimates, and findings involving this group should be interpreted with caution. Additionally, potential differences between genders may have influenced the interpretation of the findings and should be considered when evaluating the results Fourth, a single-eye analysis approach was used to avoid inter-eye correlation, with the more myopic eye selected to represent greater myopic structural changes. However, this may limit the generalizability of findings to both eyes, particularly in individuals with substantial interocular differences. Furthermore, this study is limited by the absence of key ocular covariates, including corneal curvature, lens thickness, and genetic factors. Corneal curvature is particularly critical, as the axial length to corneal radius ratio is a well-established predictor of myopia; without keratometric measurements, it was not possible to determine whether the observed refractive changes were primarily axial or corneal in origin. Likewise, the lack of lens thickness data restricts interpretation of anterior chamber depth changes, which may reflect lens thinning rather than axial elongation. These limitations may affect the interpretation of our findings and should be considered when drawing conclusions. A further limitation is that measurement reliability was not formally assessed. While all measurements were obtained by an experienced examiner, intra- and inter-observer agreement were not assessed. In addition, while automated devices such as the Pentacam reduce subjective bias, they remain susceptible to measurement variability and algorithm-related limitations (Azemin et al., 2016; Hilmi et al., 2020; Azemin et al., 2014). Future studies should evaluate the axial length-to-corneal curvature ratio to provide a more comprehensive assessment of the factors contributing to myopia. In spite of these above mentioned restrictions, the study offers innovative anterior segment biometric information in myopic eyes from this ethnic population, thereby addressing a significant gap in the existing literature and offering valuable baseline information for future research.

## Conclusions

Myopia severity in young adults is associated with significant structural changes, primarily AXL elongation and only minimal enlargement of anterior segment dimensions particularly the ACV and ACA, suggesting that the posterior expansion predominates over the anterior one. Furthermore, AXL exhibited the strongest association with refractive status, followed by ACV, CCT, and ACD, whereas ACA continued unchanging across myopia categories. Our findings are consistent with global evidence that axial elongation is the primary structural correlate of myopia, while also highlighting population-specific patterns that warrant further investigation. Clinically, this biometric profile is highly relevant for refractive surgery planning, glaucoma risk assessment, and interpretation of anterior-segment biomechanics, particularly in individuals with high myopia.

## Supplemental Information

10.7717/peerj.21767/supp-1 Supplemental Information 1 Raw Data.

10.7717/peerj.21767/supp-2 Supplemental Information 2 STROBE checklist.

## Additional Information and Declarations

### Data Availability

The following information was supplied regarding data availability:

The raw data is available in the Supplemental Files.
